# Association Between Ultrasonographic Evidence of Nerve Entrapment and Pain Outcomes After Nerve Decompression in Painful Diabetic Peripheral Neuropathy

**DOI:** 10.3390/jcm15208029

**Published:** 2026-10-16

**Authors:** Ze Zhang, Qi Wang, Zhenxuan Gao, Wenqiang Yang, Shuguang Cai, Quanyu Jin, Jiaxin Liu, Chenchen Kong, Abudurezhake Aerman, Lei Kou, Li Zhang

**Affiliations:** 1 Peking University China-Japan Friendship School of Clinical Medicine, Beijing 100029, China; ze@bjmu.edu.cn (Z.Z.); armanmed@pku.edu.cn (A.A.); 2411210694@pku.edu.cn (L.K.); 2 Department of Neurosurgery, China-Japan Friendship Hospital, Beijing 100029, China; wq2008jms@163.com (Q.W.); gaozhenxuan187@126.com (Z.G.); zrywq1985@163.com (W.Y.); 3 Department of Neurosurgery, China-Japan Friendship Hospital (Institute of Clinical Medical Sciences), Chinese Academy of Medical Sciences & Peking Union Medical College, Beijing 100029, China; csg_0227@163.com (S.C.); kccdctr@student.pumc.edu.cn (C.K.); 4 Department of Neurosurgery, Institute of Clinical Medical Sciences, China-Japan Friendship Hospital, Beijing 100029, China; liujiaxin098@163.com

**Keywords:** painful diabetic peripheral neuropathy, nerve entrapment, high-frequency ultrasonography, peripheral nerve decompression

## Abstract

**Objective:** To evaluate whether preoperative ultrasonographic evidence of nerve entrapment is associated with pain relief after lower-limb peripheral nerve decompression in patients with painful diabetic peripheral neuropathy (PDPN). **Methods:** This single-center retrospective cohort study included 110 consecutive patients with PDPN who underwent bilateral lower-limb peripheral nerve decompression between February 2023 and June 2024. After three patients were lost to follow-up, 107 were analyzed. Preoperative ultrasonography assessed the bilateral common peroneal and tibial nerves. Patients were classified as ultrasound-positive if at least one examined nerve met the specified focal entrapment criteria and as ultrasound-negative otherwise. Numeric rating scale (NRS) scores were recorded preoperatively and at 7 days, 6 months, and 12 months after surgery. A ≥50% reduction in NRS score at 12 months was defined as pain response. Analysis of covariance and multivariable logistic regression were performed. **Results:** Ultrasonographic evidence of focal entrapment in at least one examined common peroneal or tibial nerve was identified in 42 patients (39.3%). Mean NRS scores decreased from 7.9 preoperatively to 2.7, 2.5, and 2.7 at 7 days, 6 months, and 12 months, respectively (all Holm-adjusted *p* < 0.001). After adjustment for baseline NRS score, the ultrasound-positive group had lower NRS scores at 7 days and 12 months than the ultrasound-negative group; the between-group difference was not statistically significant at 6 months. At 12 months, 77 patients (72.0%) achieved pain response: 35 of 42 ultrasound-positive patients (83.3%) and 42 of 65 ultrasound-negative patients (64.6%). Ultrasound-positive status was associated with higher odds of pain response after multivariable adjustment (adjusted odds ratio [OR], 3.69; 95% confidence interval [CI], 1.28–10.67; *p* = 0.016). Firth bias-reduced logistic regression yielded a similar estimate (OR, 3.32; 95% profile-likelihood CI, 1.26–9.81; *p* = 0.015). **Conclusions:** Preoperative ultrasonographic evidence of nerve entrapment was associated with more favorable postoperative pain outcomes after nerve decompression in patients with PDPN. Its potential prognostic value requires prospective validation.

## 1. Introduction

The increasing prevalence of diabetes has led to a growing disease burden associated with diabetic peripheral neuropathy (DPN) [1]. A proportion of patients with DPN develop persistent or recurrent neuropathic pain, typically characterized by burning, stabbing, electric shock-like, or tingling sensations. This condition is known as painful diabetic peripheral neuropathy (PDPN) [2]. PDPN can substantially impair mobility, sleep, emotional well-being, and overall quality of life. Pharmacological therapy remains the mainstay of treatment; however, some patients experience inadequate pain relief or intolerable adverse effects [3]. Moreover, pharmacotherapy generally does not reverse established structural damage to peripheral nerves [4]. These limitations have prompted interest in potentially modifiable contributors to persistent pain.

PDPN and focal entrapment neuropathy are distinct but potentially coexisting processes. PDPN typically causes bilateral, length-dependent pain associated with diffuse peripheral nerve injury, whereas entrapment neuropathy involves mechanical compromise of a specific nerve segment. Diabetes-related metabolic disturbances, microvascular dysfunction, intraneural edema, and nerve enlargement may increase susceptibility to compression at anatomically constrained sites, such as the fibular and tarsal tunnels [5,6]. This combination of diffuse metabolic injury and superimposed focal compression underlies the double-crush concept in diabetic neuropathy. When focal compression contributes to pain, anatomical concordance between pain or sensory disturbance and focal neurological or provocative findings in the corresponding nerve territory would be expected. Persistent compression may impair intraneural perfusion and axonal conduction, promote ectopic neural activity, and contribute to peripheral and central sensitization [6].

Peripheral nerve decompression has been proposed for selected patients with PDPN to relieve a potentially modifiable mechanical component of nerve injury. Previous studies have reported improvements in pain, sensory function, nerve conduction, and quality of life [7,8,9]. However, patient selection has varied, with different combinations of pain distribution, the Tinel sign, sensory examination, two-point discrimination, electrophysiological testing, and imaging used to assess focal nerve involvement [7,8,9,10]. Consequently, surgery may target potential entrapment sites without preoperative confirmation of focal structural compression. Postoperative outcomes also vary, highlighting the need to identify preoperative features associated with sustained, clinically meaningful pain relief.

High-frequency nerve ultrasonography is noninvasive and provides structural information that complements clinical and neurological assessment [11]. Previous studies have primarily examined nerve morphology and postoperative structural changes. Patients with diabetic neuropathy may have enlarged peripheral nerves, including increased tibial nerve cross-sectional area at the tarsal tunnel [12,13]. Longitudinal studies have reported changes in nerve morphology alongside sensory and electrophysiological improvements after decompression [14,15]. However, structural findings have been inconsistent: in a prospective randomized study, Macaré van Maurik et al. found no significant difference in the change in tibial nerve cross-sectional area between decompressed and contralateral control limbs [13]. Outcome stratification has also relied mainly on clinical features, such as pain distribution [15]. Whether preoperative focal ultrasonographic entrapment findings are associated with postoperative pain outcomes, and whether they add prognostic information beyond clinical and electrophysiological assessment, remains unclear.

Clinical assessment of potential entrapment commonly includes symptoms and signs such as the Tinel sign [10,16,17], whereas high-frequency ultrasonography provides structural information about the examined nerve segments. Accordingly, the present study investigated the association between preoperative ultrasonographic evidence of common peroneal or tibial nerve entrapment and postoperative pain outcomes in patients with PDPN undergoing bilateral lower-limb peripheral nerve decompression.

## 2. Materials and Methods

### 2.1. Patients

We retrospectively identified 110 consecutive patients with PDPN who underwent bilateral lower-limb peripheral nerve decompression at China–Japan Friendship Hospital between February 10, 2023 and June 26, 2024. DPN was diagnosed clinically using established criteria [18,19]: diabetes mellitus with compatible distal, predominantly symmetrical sensory symptoms and/or neurological signs, after consideration of alternative causes of polyneuropathy. Assessment included the distribution and characteristics of sensory symptoms and a lower-limb neurological examination. PDPN was defined as DPN accompanied by persistent bilateral distal lower-limb neuropathic pain, typically burning, stabbing, electric shock-like, or tingling, with a Douleur Neuropathique 4 (DN4) score ≥ 4. The DN4 questionnaire supported the identification of neuropathic pain; it was not used to establish the diagnosis of DPN.

Patients were included if they met all of the following criteria:

(1) age ≥ 18 years;

(2) a clinical diagnosis of diabetes mellitus and DPN;

(3) persistent bilateral distal lower-limb neuropathic pain with an NRS score ≥ 5;

(4) adequate lower-limb perfusion, defined as an ankle–brachial index (ABI) ≥ 0.90;

(5) inadequate pain control despite guideline-recommended pharmacological treatment;

(6) completion of preoperative high-frequency ultrasonography of the lower-limb peripheral nerves.

The study protocol was approved by the Clinical Research Ethics Committee of China–Japan Friendship Hospital (approval No. 2026-KY-054). Given the retrospective design of the study and the use of anonymized patient data, the requirement for written informed consent was waived by the ethics committee. The study was conducted in accordance with the Declaration of Helsinki.

### 2.2. Ultrasonographic Examination and Definition of Nerve Entrapment

Preoperative ultrasonographic examinations were performed using a Philips EPIQ 7 ultrasound system (Philips Healthcare, Inc., Bothell, WA, USA) equipped with a 4–18 MHz high-frequency linear-array transducer. Bilateral common peroneal and tibial nerves were continuously examined in both transverse and longitudinal planes. Patient positioning was adjusted according to the anatomical course of the target nerve. Gain, imaging depth, and focal-zone settings were optimized to clearly visualize the fascicular architecture. Minimal transducer pressure was applied to avoid compression-induced deformation of the nerve. For examination of the common peroneal nerve, patients were positioned supine or in the lateral decubitus position with the knee slightly flexed. After identification of the nerve at the lateral aspect of the popliteal fossa, it was continuously traced distally to the fibular head and fibular neck. For examination of the tibial nerve, patients were positioned supine with the lower limb externally rotated. The nerve was identified at the posteromedial aspect of the lower leg and traced distally to the tarsal tunnel posterior to the medial malleolus and its distal branches. Morphology at potential entrapment sites was compared with adjacent segments, focusing on caliber, echogenicity, fascicular architecture, and relationships with surrounding structures.

**Figure 1:**
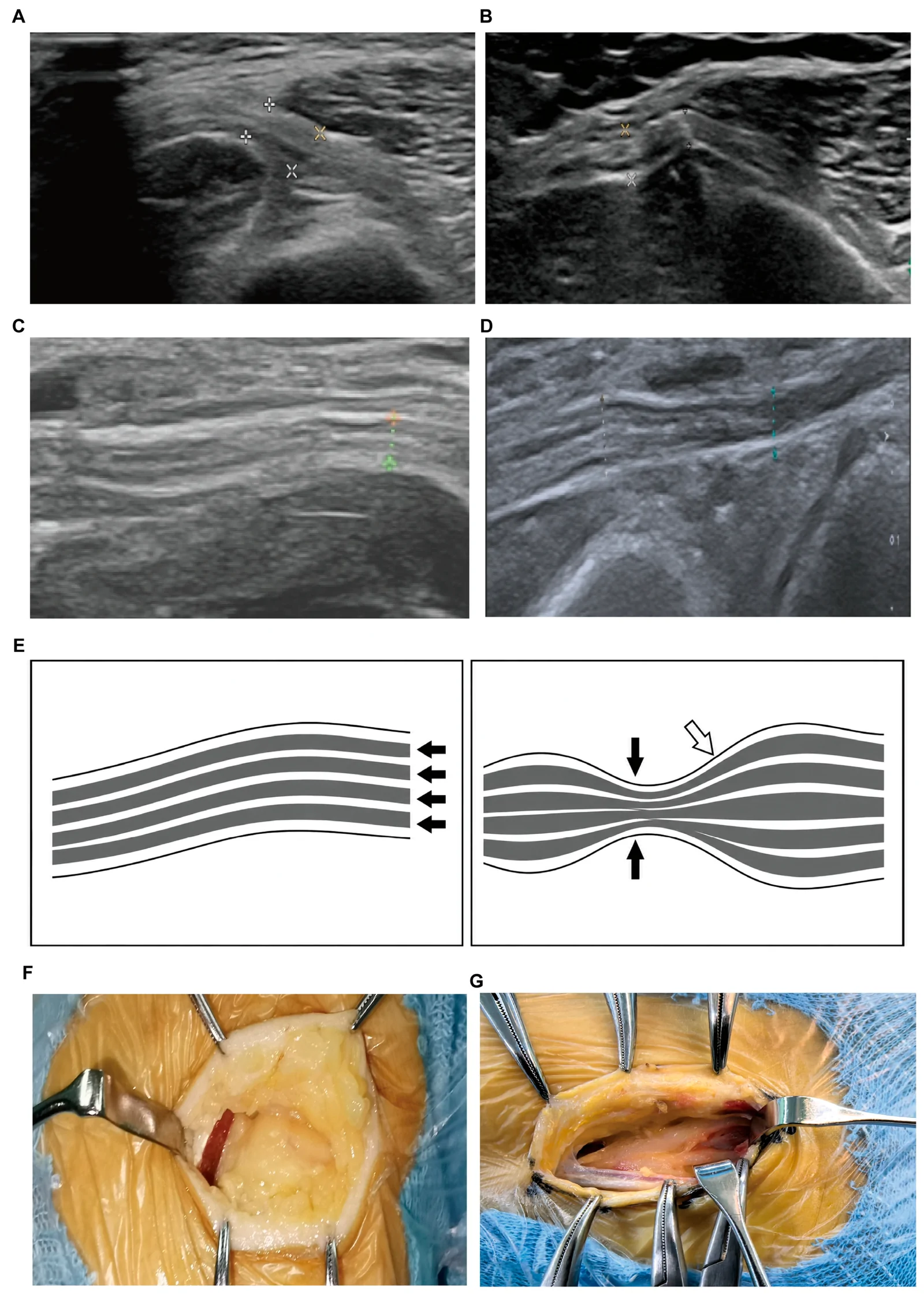
Representative findings of common peroneal nerve and tibial nerve entrapment. (**A**) Normal common peroneal nerve at the fibular tunnel. (**B**) Entrapment of the common peroneal nerve at the fibular tunnel. (**C**) Normal tibial nerve at the tarsal tunnel. (**D**) Entrapment of the tibial nerve at the tarsal tunnel. (**E**) Schematic illustration of normal and entrapped peripheral nerves. Arrows indicate the structural features of normal and compressed nerves. (**F**) Intraoperative image showing common peroneal nerve entrapment at the fibular tunnel. (**G**) Intraoperative image showing tibial nerve entrapment at the tarsal tunnel.

Entrapment was classified using a qualitative morphological framework. Definite ultrasonographic entrapment required focal compression or flattening at an anatomically plausible site plus at least one supportive feature: (1) proximal focal enlargement, (2) an abrupt change in caliber across the suspected site, or (3) direct visualization of nerve compression within a fibro-osseous tunnel on longitudinal imaging. Diffuse enlargement, reduced echogenicity, or altered fascicular architecture alone did not qualify. Patients were classified as ultrasound-positive if at least one common peroneal or tibial nerve on either side met these criteria and as ultrasound-negative otherwise, including examinations with isolated or borderline abnormalities. This classification applied only to the examined nerves, not to all surgically decompressed sites. The classification criteria were established before postoperative outcomes were reviewed. Two investigators (Z.Z. and Q.W.), blinded to postoperative outcomes, independently reviewed the classification; disagreements were resolved by discussion or adjudication by a senior investigator (L.Z.). Cross-sectional area, swelling ratio, and flattening ratio were not systematically recorded and were not used for classification. Representative findings are shown in Figure 1.

### 2.3. Surgical Procedure

All patients underwent standardized bilateral lower-limb peripheral nerve decompression under general anesthesia. The procedure involved decompression of common sites of nerve entrapment in the lower limbs, including the common peroneal nerve at the fibular head, the tibial nerve and its branches at the medial ankle, the deep peroneal nerve between the first and second metatarsals on the dorsum of the foot, and the superficial peroneal nerve at the junction of the middle and distal thirds of the lower leg.

Fascial structures and fibrous bands potentially compressing the nerves were divided. Local anesthetic infiltration and peripheral nerve blocks were not routinely used for postoperative analgesia. Perioperative care included glycemic control, antimicrobial prophylaxis, multimodal analgesia, wound care, and early rehabilitation. The target fasting blood glucose range was 6.2–8.0 mmol/L. Intravenous broad-spectrum antibiotics were administered 30–60 min before surgery. Non-opioid analgesics and adjunctive measures were provided as needed for surgical-site pain, separately from chronic PDPN medications. Analgesic classes, doses, treatment duration, and rescue use were not systematically recorded. Gentle ankle range-of-motion exercises began on postoperative day 2, and mobilization with crutch assistance was permitted on day 3. Sutures were generally removed on day 14, depending on wound healing. The distal peroneal decompression sites were not systematically included in the preoperative ultrasonographic assessment. Therefore, ultrasound-negative status referred only to the examined common peroneal and tibial nerves and did not exclude compression at other surgically decompressed sites.

### 2.4. Clinical Data Collection

Collected variables included age, sex, body mass index (BMI), diabetes duration, glycated hemoglobin, fasting and postprandial plasma glucose, lipid profiles, hypertension, smoking, alcohol use, foot ulcer history, and diabetic kidney disease. Neutrophil and lymphocyte counts were recorded to calculate the neutrophil-to-lymphocyte ratio. Adverse events and surgical complications were not systematically collected using predefined definitions or standardized surveillance; therefore, complication rates could not be reliably estimated and no formal safety analysis was performed.

### 2.5. Pain Assessment and Outcome Measures

Pain was assessed using an 11-point NRS, from 0 (no pain) to 10 (worst imaginable pain) [20]. Patients provided a single score for their current overall bilateral distal lower-limb pain preoperatively and at 7 days, 6 months, and 12 months after surgery. The 6- and 12-month assessments were conducted during outpatient visits or by telephone.

When clinically feasible, patients taking symptomatic PDPN medications were instructed to withhold them for 3 days before each scheduled pain assessment. This pragmatic interval aimed to reduce short-term medication effects but did not represent a drug-specific pharmacokinetic washout. Adherence was self-reported and not objectively verified.

The primary outcome was the 12-month postoperative NRS score, compared between the ultrasound-positive and ultrasound-negative groups. Secondary outcomes included NRS scores at 7 days and 6 months and changes from baseline at each follow-up. Patients with a ≥50% reduction in NRS score at 12 months were classified as responders; those with a smaller reduction were classified as nonresponders. This patient-level response criterion is distinct from the interpretation of between-group differences [21].

### 2.6. Statistical Analysis

Continuous variables are reported as mean (standard deviation [SD]) or median [interquartile range (IQR)] and were compared using Student’s *t*-test, Welch’s *t*-test, or the Mann–Whitney U test, as appropriate. Categorical variables are reported as *n* (%) and were compared using the chi-square test or Fisher’s exact test. Analyses used Python 3.13.5, statsmodels 0.14.6, and SciPy 1.17.0. All tests were two-sided, with *p* < 0.05 considered statistically significant.

A one-way repeated-measures analysis of variance compared NRS scores across baseline and the three postoperative assessments. The Greenhouse–Geisser correction was applied when sphericity was violated. Following a significant overall time effect, paired baseline-to-follow-up comparisons were performed, with Holm adjustment across the three comparisons.

Analysis of covariance (ANCOVA) compared postoperative NRS scores between groups at each follow-up, with ultrasonographic status as the fixed factor and baseline NRS score as the covariate. Homogeneity of regression slopes was assessed using the interaction between ultrasonographic status and baseline NRS score. Residuals and variance assumptions were examined, and HC3 heteroscedasticity-robust standard errors were used for inference. The three adjusted between-group contrasts formed a separate family of tests for Holm adjustment. The reported 95% confidence intervals (CIs) are nominal; nominal and Holm-adjusted *p* values are distinguished where applicable.

Univariable and multivariable logistic regression evaluated associations with 12-month pain response. Maximum-likelihood models yielded crude and adjusted odds ratios (ORs) with Wald 95% CIs. The multivariable model included ultrasonographic status, age, sex, diabetes duration, and baseline NRS score. Pain response, ultrasound-positive status, and female sex were coded as 1; their respective reference categories were coded as 0. Age, diabetes duration, and baseline NRS score were continuous variables. All 107 patients were included.

Given the limited number of nonresponders, Firth bias-reduced logistic regression with the same covariates was used for sensitivity analysis. ORs were estimated by penalized likelihood, with penalized profile-likelihood 95% CIs and penalized likelihood-ratio *p* values. Model convergence, separation, and influential observations were assessed, including leave-one-out Firth analyses.

## 3. Results

### 3.1. Baseline Characteristics

**Figure 2:**
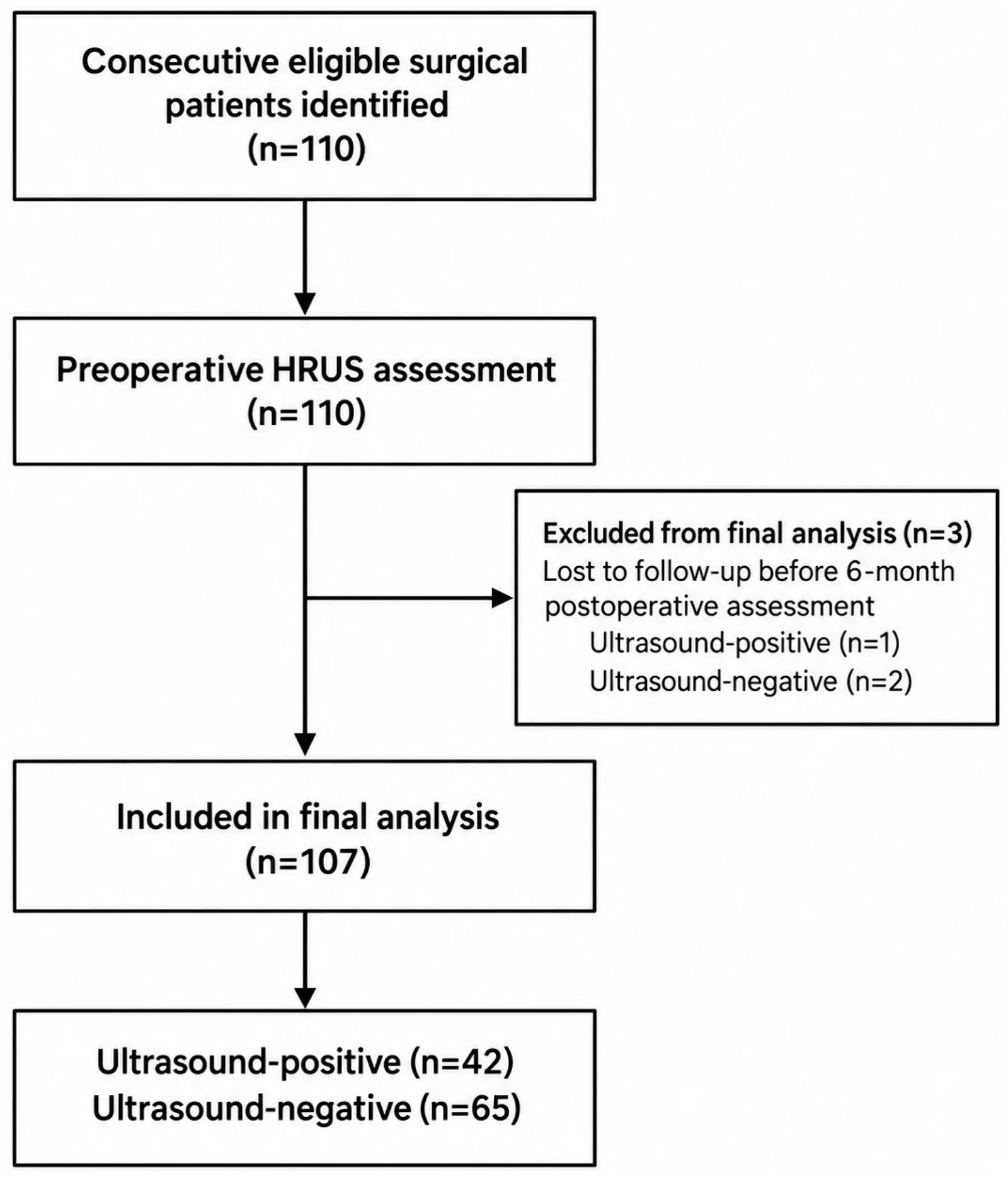
Flow of the identified surgical cohort and final analysis population. HRUS, high-resolution ultrasonography. The diagram begins with the eligible surgical cohort and does not enumerate all patients considered for decompression.

Three of the 110 patients were lost to follow-up before the 6-month assessment and excluded, leaving 107 for analysis. Of the three excluded patients, one was ultrasound-positive and two were ultrasound-negative. Figure 2 shows the patient-selection process. The 110 patients constituted the identified surgical cohort before exclusion for incomplete follow-up.

Of the 107 patients analyzed, 42 (39.3%) were ultrasound-positive and 65 (60.7%) were ultrasound-negative. Mean age was 62.2 years (SD 9.8); 69 patients (64.5%) were men and 38 (35.5%) were women. Mean BMI was 24.4 kg/m^2^ (SD 2.9), and median diabetes duration was 10.0 years (IQR 6.0–20.0).

No statistically significant between-group differences were observed in demographic characteristics, diabetes-related variables, vascular risk factors, lipid profiles, or hematological parameters (all *p* > 0.05; Table 1).

Preoperative NRS scores were higher in the ultrasound-positive group (mean 8.4 [SD 1.1]) than in the ultrasound-negative group (7.6 [SD 1.2]; *p* < 0.001; Table 1).

### 3.2. Distribution of Ultrasonographic Entrapment Findings

Among the 42 ultrasound-positive patients, the common peroneal and tibial nerves were each affected in 30 patients (71.4%). Entrapment involved only the common peroneal nerve in 12 patients (28.6%), only the tibial nerve in 12 (28.6%), and both nerve types in 18 (42.9%).

Entrapment findings were unilateral in 10 patients (23.8%) and bilateral in 32 (76.2%). Counting each nerve on each side separately, 9 patients (21.4%) had one affected nerve, 17 (40.5%) had two, 1 (2.4%) had three, and 15 (35.7%) had all four examined nerves affected.

### 3.3. Changes in Pain Scores After Nerve Decompression

**Figure 3:**
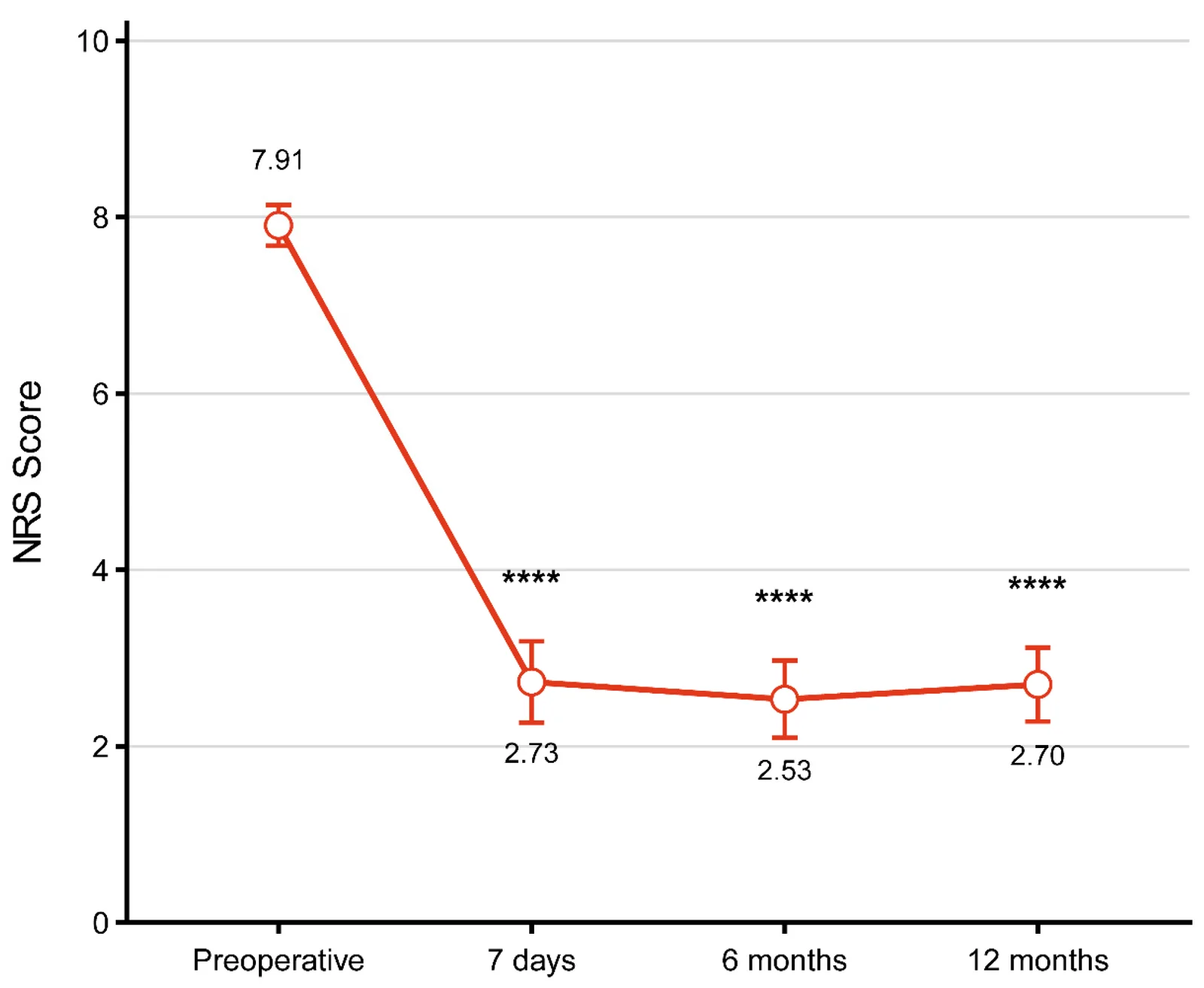
NRS scores before and after bilateral lower-limb peripheral nerve decompression. Values are means with 95% CIs. Each postoperative comparison with baseline had a Holm-adjusted *p* < 0.001. The asterisks (****) indicate Holm-adjusted *p* < 0.001 versus baseline. NRS, numeric rating scale; CI, confidence interval.

All 107 patients had complete NRS data at the analyzed time points. Mean NRS scores decreased from 7.9 preoperatively to 2.7 at 7 days, 2.5 at 6 months, and 2.7 at 12 months. Mauchly’s test indicated a violation of sphericity (W = 0.808, *p* < 0.001), so the Greenhouse–Geisser correction was applied (ε = 0.894). The overall time effect was significant (F [2.68, 284.17] = 182.93, *p* < 0.001), and each postoperative score differed from baseline after Holm adjustment (all adjusted *p* < 0.001; Figure 3).

### 3.4. Baseline-Adjusted Postoperative Pain Outcomes

ANCOVA accounted for the higher baseline NRS scores in the ultrasound-positive group. Interactions between ultrasonographic status and baseline NRS score were not statistically significant at 7 days, 6 months, or 12 months (*p* = 0.708, *p* = 0.437, and *p* = 0.549, respectively), providing no evidence against homogeneity of regression slopes. Residual plots showed departures from normality for the bounded NRS outcome. Breusch–Pagan tests indicated heteroscedasticity at 7 days (*p* = 0.003) and 12 months (*p* = 0.017), but not at 6 months (*p* = 0.352). HC3 heteroscedasticity-robust standard errors were therefore used at all three time points.

Baseline-adjusted NRS scores were lower in the ultrasound-positive group at 7 days (adjusted mean difference, −1.53; nominal 95% CI, −2.50 to −0.56; nominal *p* = 0.002; Holm-adjusted *p* = 0.004) and 12 months (−1.65; nominal 95% CI, −2.54 to −0.76; Holm-adjusted *p* = 0.001). The 6-month difference was not statistically significant (−0.87; nominal 95% CI, −1.83 to 0.08; Holm-adjusted *p* = 0.073; Table 2).

### 3.5. Characteristics of Responders and Nonresponders

At 12 months, 77 patients (72.0%) were responders and 30 (28.0%) were nonresponders. No statistically significant differences were observed in the demographic, clinical, or laboratory variables listed in Table 3, including baseline NRS score (all *p* > 0.05).

Ultrasonographic evidence of entrapment was more common among responders than nonresponders (45.5% vs. 23.3%; *p* = 0.047; Table 3).

### 3.6. Factors Associated with Pain Response at 12 Months

In univariable logistic regression, ultrasonographic evidence of entrapment was associated with higher odds of 12-month pain response (OR, 2.74; 95% CI, 1.05–7.13; *p* = 0.039).

After adjustment for age, sex, diabetes duration, and baseline NRS score, ultrasound-positive status remained associated with pain response (adjusted OR, 3.69; 95% CI, 1.28–10.67; *p* = 0.016). The other covariates were not significantly associated with response (all *p* > 0.05; Table 4).

Firth bias-reduced logistic regression yielded a similar association (adjusted OR, 3.32; 95% profile-likelihood CI, 1.26–9.81; *p* = 0.015). The conventional model converged with finite estimates and no evidence of complete separation. Although some observations had greater influence, leave-one-out Firth estimates for ultrasound-positive status ranged from 3.01 to 4.58. The direction of the association was therefore consistent across these analyses, although its magnitude remained imprecisely estimated.

## 4. Discussion

In this retrospective cohort of patients with refractory PDPN undergoing bilateral lower-limb nerve decompression, preoperative ultrasonographic evidence of focal common peroneal or tibial nerve entrapment was associated with more favorable pain outcomes at 12 months. This association was evident in both baseline-adjusted pain scores and the proportion achieving a ≥50% reduction in pain, and its direction was consistent after multivariable adjustment and Firth sensitivity analysis. The standardized surgical approach and availability of 12-month outcomes provided an opportunity to examine outcome heterogeneity within a relatively consistent treatment setting. These findings suggest that focal ultrasonographic abnormalities may identify a clinically relevant phenotype within PDPN and support further evaluation of ultrasound as a complementary component of preoperative assessment.

The distinction between diffuse diabetic polyneuropathy and superimposed focal entrapment provides a biological framework for interpreting this association. Diabetes-related metabolic and microvascular abnormalities may increase nerve vulnerability to mechanical stress at anatomically constrained sites [6]. In this setting, a focal lesion may contribute to symptoms alongside the underlying diffuse neuropathy. Our ultrasound criteria emphasized focal flattening accompanied by supportive segmental changes, rather than diffuse enlargement alone, to characterize this possible mechanical component. Decompression could plausibly improve intraneural perfusion and venous outflow and reduce ischemia or ectopic neural activity. Although these mechanisms were not directly measured, they provide hypotheses linking focal morphology to postoperative pain outcomes. Their evaluation would benefit from demonstrating anatomical concordance among imaging abnormalities, pain distribution, focal neurological findings, and electrophysiological dysfunction.

The present findings extend earlier investigations of ultrasound morphology in diabetic neuropathy. Lee and Dauphinée explored ultrasound together with neurosensory testing in the assessment of potential decompression candidates [12]. In a prospective randomized study, Macaré van Maurik et al. found no significant difference in tibial nerve cross-sectional area (CSA) change between decompressed and contralateral control limbs [13]. This finding cautions against assuming that decompression necessarily reverses diabetic nerve enlargement. Postoperative CSA change and the prognostic relevance of preoperative focal morphology nevertheless address different questions. Our study contributes to the latter by relating a focal ultrasound phenotype to subsequent pain outcomes. Quantitative morphometry remains essential for further characterization of this phenotype: standardized CSA measurements, swelling ratios, and flattening ratios would allow focal geometry to be evaluated alongside overall nerve size and improve comparability across studies.

The agreement between continuous and responder outcomes supports the potential clinical relevance of the observed association. At 12 months, ultrasound-positive patients had an adjusted NRS score 1.65 points lower than ultrasound-negative patients (nominal 95% CI for the difference, −2.54 to −0.76), and response rates were 83.3% and 64.6%, respectively. Ultrasound positivity was associated with higher adjusted odds of response (OR, 3.69; 95% CI, 1.28–10.67), with a similar estimate in the Firth analysis (OR, 3.32; 95% profile-likelihood CI, 1.26–9.81). The mean difference suggests potentially meaningful separation in postoperative pain burden, although its clinical importance requires consideration alongside functional recovery, quality of life, and safety. A between-group mean difference and the individual criterion of ≥50% pain reduction describe distinct aspects of outcome and should be interpreted separately [21]. At 6 months, the adjusted difference favored the ultrasound-positive group but remained inconclusive (−0.87 points; nominal 95% CI, −1.83 to 0.08). Differences in statistical significance across visits do not establish a changing association over time; this would require a formal group-by-time analysis.

The substantial improvement among ultrasound-negative patients provides additional insight into the interpretation of the imaging phenotype. Forty-two of 65 ultrasound-negative patients achieved the response criterion, accounting for 54.5% of all responders. Ultrasound-negative status indicated the absence of definite focal abnormalities under the present criteria, rather than the absence of a mechanical contribution to symptoms. Diabetes-related nerve enlargement provides a background in which superimposed focal abnormalities must be distinguished from more widespread morphological changes [6,12,22]. One possibility is that diffuse enlargement alters the contrast between adjacent nerve segments, making subtle focal caliber changes less conspicuous on qualitative assessment. This explanation remains to be tested with standardized segmental measurements. Borderline abnormalities classified as negative and compression at superficial or deep peroneal sites outside the ultrasound protocol could also contribute to the observed response. Thus, criteria requiring visible focal deformation may not capture the full spectrum of mechanically relevant nerve involvement. Postoperative improvement alone, however, cannot establish occult compression, because concurrent treatment, symptom variability, and contextual effects may also influence pain. These findings favor evaluating ultrasound as complementary information within a multimodal assessment rather than as an exclusionary criterion for surgery.

This complementary role is consistent with the different information provided by imaging, clinical examination, and electrophysiological assessment. Ultrasound depicts nerve morphology and its relationship to surrounding structures, whereas pain distribution, provocative signs, sensory testing, and nerve conduction studies characterize the clinical and functional expression of nerve injury. Previous studies have examined clinical indicators such as the Tinel sign and two-point discrimination in relation to decompression outcomes [10,16,17]. Integrating these domains may help distinguish a clinically concordant focal lesion from an incidental structural abnormality. The current study provides a rationale for evaluating such integration, although the incremental prognostic contribution of ultrasound beyond routine clinical and electrophysiological measures remains to be established.

These implications should also be considered within the evolving evidence for decompression in PDPN. Observational studies and systematic reviews have reported postoperative improvements, with variation in study design, selection criteria, and follow-up [23,24,25]. Decompression for a clinically established focal entrapment neuropathy and multilevel decompression for diffuse PDPN address different clinical indications. In 2006, the American Academy of Neurology classified decompression for diabetic neuropathy as unproven (Level U), based on the Class IV evidence then available [26]. The advisory was retired in 2024 and is now a historical reference [27]. More recent controlled evidence includes the trial by Rozen et al., in which decompressed and sham-operated limbs improved similarly at 12 months, whereas outcomes favored decompressed limbs at 56 months [28]. These results emphasize the importance of controlled comparisons and follow-up duration when evaluating surgical effects. Our study addresses the related question of which preoperative features are associated with outcomes among surgically treated patients. Determining whether the ultrasound phenotype identifies additional benefit specifically attributable to decompression requires a controlled comparison with a prespecified ultrasound-by-treatment interaction.

Several limitations merit consideration. The single-center retrospective design is susceptible to selection bias and residual confounding. Adjustment for baseline pain and sensitivity analyses strengthen the interpretation of the observed association but cannot account for unmeasured differences in neuropathy severity, symptom duration, expectations, or concurrent treatment. Diabetes duration is not a substitute for neuropathy duration or severity. Moreover, the absence of a nonsurgical comparator means that the same phenotype might also be associated with improvement during optimized medical treatment or rehabilitation. Only 30 patients were nonresponders, including seven in the ultrasound-positive group, providing limited information for a model with five predictor coefficients. The wide confidence intervals therefore warrant caution when interpreting effect magnitude, and the analyses do not establish individual predictive performance. Exclusion of three patients with incomplete follow-up may introduce attrition bias. Although treatment by one senior surgeon improved procedural consistency, reproducibility across centers, surgeons, and ultrasound operators remains to be established.

The imaging and outcome definitions introduce additional limitations. CSA, swelling ratio, and flattening ratio were not systematically recorded, leaving no quantitative thresholds for reproducing the classification. Acquisition technique, anatomical variation, and reader judgment may have influenced group assignment, particularly for borderline findings. Independent review and adjudication improved consistency but did not provide a formal estimate of interobserver or intraobserver reliability. In addition, positivity in any examined nerve determined patient-level status, whereas pain was recorded as a single current overall bilateral NRS score. This approach combined abnormalities of different distributions and prevented assessment of whether improvement occurred in the territory of an ultrasound-positive nerve. The superficial and deep peroneal surgical sites were not systematically examined; consequently, ultrasound-negative status applies only to the assessed nerves. Longitudinal analgesic exposure was incompletely documented, and the 3-day medication withholding interval was neither drug-specific nor objectively verified. These factors, together with possible overlap between incisional and neuropathic pain, particularly affect interpretation of the day-7 assessment. The absence of systematic adverse-event surveillance also limits evaluation of surgical safety.

Prospective multicenter studies should determine whether this association can be reproduced and translated into useful clinical information. Standardized quantitative imaging, blinded interpretation, reliability testing, and annotated positive, negative, and equivocal examinations would improve measurement consistency. Nerve- and side-specific findings should be linked to symptom distribution and clinical and electrodiagnostic measures of neuropathy severity. Controlled studies incorporating optimized nonsurgical care and, where ethically justified, sham surgery with superficial exposure without nerve release would help distinguish general prognostic relevance from treatment-specific benefit. Blinding should include participants, outcome assessors, and analysts where feasible, with independent ultrasound interpretation. Longitudinal mixed-effects models could evaluate outcome trajectories while accounting for repeated observations and clustering within patients. Pain outcomes should be accompanied by functional, electrophysiological, medication-use, and systematically collected safety measures. External validation and comparison with routine clinical assessment will be needed to establish incremental prognostic value. The present findings provide a basis for this work by identifying a preoperative structural phenotype associated with postoperative pain outcomes in PDPN.

## 5. Conclusions

Preoperative ultrasonographic evidence of common peroneal or tibial nerve entrapment was associated with lower baseline-adjusted NRS scores at 7 days and 12 months and higher odds of 12-month pain response after bilateral lower-limb nerve decompression in patients with PDPN. These exploratory findings support further evaluation of ultrasonography for postoperative prognostic assessment. Reproducibility, incremental clinical value, and individual predictive performance require validation in larger prospective multicenter studies.

## Figures and Tables

**Table 1:** Baseline characteristics.

Characteristic	Overall *n* = 107	Ultrasound-Negative *n* = 65	Ultrasound-Positive *n* = 42	*p* Value
Age, years	62.2 (9.8)	61.8 (10.4)	62.8 (8.9)	0.598
Sex				0.301
Male	69 (64.5%)	39 (60.0%)	30 (71.4%)	
Female	38 (35.5%)	26 (40.0%)	12 (28.6%)	
BMI, kg/m^2^	24.4 (2.9)	24.6 (2.9)	24.1 (2.9)	0.398
Diabetes duration, years	10.0 [6.0, 20.0]	12.0 [7.0, 18.0]	10.0 [4.0, 20.0]	0.481
Hypertension	57 (53.3%)	36 (55.4%)	21 (50.0%)	0.692
Smoking	11 (10.3%)	7 (10.8%)	4 (9.5%)	>0.999
Alcohol use	14 (13.1%)	11 (16.9%)	3 (7.1%)	0.240
History of foot ulcer	6 (5.6%)	4 (6.2%)	2 (4.8%)	>0.999
Diabetic kidney disease	6 (5.6%)	4 (6.2%)	2 (4.8%)	>0.999
HbA1c, %	7.5 (2.4)	7.5 (2.6)	7.5 (2.1)	0.981
Fasting plasma glucose, mmol/L	6.2 [5.4, 8.1]	6.1 [5.2, 7.9]	6.3 [5.5, 8.8]	0.434
Postprandial glucose, mmol/L	10.1 [8.1, 12.5]	10.2 [8.2, 11.8]	10.0 [7.7, 13.1]	0.972
Total cholesterol, mmol/L	4.5 (1.3)	4.5 (1.2)	4.6 (1.5)	0.612
Triglycerides, mmol/L	1.5 [1.0, 2.2]	1.5 [1.0, 2.2]	1.3 [0.9, 2.5]	0.577
HDL-C, mmol/L	1.4 (0.5)	1.4 (0.5)	1.3 (0.4)	0.218
LDL-C, mmol/L	2.4 (0.9)	2.4 (0.9)	2.5 (1.0)	0.737
Neutrophil count, ×10^9^/L	3.9 (1.8)	4.0 (1.8)	3.9 (1.6)	0.896
Lymphocyte count, ×10^9^/L	1.9 (0.9)	2.0 (1.0)	1.8 (0.6)	0.251
Neutrophil-to-lymphocyte ratio	2.0 [1.5, 2.6]	2.1 [1.5, 2.6]	2.0 [1.6, 2.5]	0.730
Preoperative NRS score	7.9 (1.2)	7.6 (1.2)	8.4 (1.1)	<0.001

Note: Values are mean (SD), median [IQR], or *n* (%). BMI, body mass index; NRS, numeric rating scale. SD, standard deviation; IQR, interquartile range; HbA1c, glycated hemoglobin; HDL-C, high-density lipoprotein cholesterol; LDL-C, low-density lipoprotein cholesterol.

**Table 2:** Comparison of postoperative NRS scores between the ultrasound-positive and ultrasound-negative groups after adjustment for preoperative NRS scores.

Follow-Up Time	Ultrasound- Negative Adjusted Mean (Nominal 95% CI)	Ultrasound- Positive Adjusted Mean (Nominal 95% CI)	Adjusted Mean Difference (Nominal 95% CI)	Nominal *p* Value	Holm-Adjusted *p* Value	Interaction *p* Value
7 days	3.33 (2.67–3.99)	1.80 (1.16–2.44)	−1.53 (−2.50 to −0.56)	0.002	0.004	0.708
6 months	2.88 (2.28–3.47)	2.00 (1.30–2.70)	−0.87 (−1.83 to 0.08)	0.073	0.073	0.437
12 months	3.35 (2.80–3.90)	1.70 (1.06–2.34)	−1.65 (−2.54 to −0.76)	<0.001	0.001	0.549

Note: Adjusted means were estimated after adjustment for preoperative NRS score. Standard errors and nominal 95% CIs were calculated using the HC3 heteroscedasticity-consistent covariance estimator. Holm adjustment was applied across the three between-group contrasts at 7 days, 6 months, and 12 months. Confidence intervals and interaction *p* values were not multiplicity-adjusted. Adjusted differences are ultrasound-positive minus ultrasound-negative. Interaction *p* values refer to the baseline-NRS-by-ultrasound-status interaction. NRS, numeric rating scale; CI, confidence interval; HC3, heteroscedasticity-consistent covariance estimator type 3.

**Table 3:** Characteristics of responders and nonresponders.

Characteristic	Nonresponder *n* = 30	Responder *n* = 77	*p* Value
Age, years	63.0 [53.0, 69.0]	64.0 [59.0, 67.0]	0.581
Sex			0.507
Male	21 (70.0%)	48 (62.3%)	
Female	9 (30.0%)	29 (37.7%)	
BMI, kg/m^2^	24.5 (2.7)	24.3 (3.0)	0.817
Diabetes duration, years	10.0 [6.0, 20.0]	11.0 [6.0, 18.0]	0.961
Hypertension	14 (46.7%)	43 (55.8%)	0.518
Smoking	1 (3.3%)	10 (13.0%)	0.176
Alcohol use	4 (13.3%)	10 (13.0%)	>0.999
History of foot ulcer	2 (6.7%)	4 (5.2%)	0.672
Diabetic kidney disease	0 (0.0%)	6 (7.8%)	0.182
HbA1c, %	6.7 [6.1, 7.6]	6.8 [6.2, 8.1]	0.407
Fasting plasma glucose, mmol/L	6.5 [5.5, 8.1]	6.1 [5.4, 8.0]	0.693
Postprandial glucose, mmol/L	10.6 [8.8, 12.6]	9.8 [7.7, 12.3]	0.337
Total cholesterol, mmol/L	4.3 [3.9, 5.5]	4.2 [3.5, 4.9]	0.230
Triglycerides, mmol/L	1.4 [1.0, 1.7]	1.5 [0.9, 2.4]	0.779
HDL-C, mmol/L	1.2 [1.1, 1.7]	1.3 [1.0, 1.6]	0.906
LDL-C, mmol/L	2.2 [1.6, 2.7]	2.3 [1.8, 3.1]	0.353
Neutrophil count, ×10^9^/L	3.3 [2.9, 4.7]	3.6 [2.9, 4.5]	0.517
Lymphocyte count, ×10^9^/L	1.8 [1.5, 2.2]	1.9 [1.5, 2.2]	0.750
Neutrophil-to-lymphocyte ratio	2.4 [1.3, 2.7]	2.0 [1.5, 2.5]	0.784
Preoperative NRS score	8.0 (1.2)	7.9 (1.2)	0.490
Ultrasonographic evidence of nerve entrapment	7 (23.3%)	35 (45.5%)	0.047

Note: Values are mean (SD), median [IQR], or *n* (%). SD, standard deviation; IQR, interquartile range; BMI, body mass index; HbA1c, glycated hemoglobin; HDL-C, high-density lipoprotein cholesterol; LDL-C, low-density lipoprotein cholesterol; NRS, numeric rating scale.

**Table 4:** Logistic regression analyses of factors associated with pain response at 12 months.

Variable	Crude OR (95% CI)	*p* Value	Conventional Adjusted OR (95% CI)	*p* Value	Firth-Adjusted OR (95% Profile-Likelihood CI)	*p* Value
Ultrasonographic evidence of nerve entrapment	2.74 (1.05–7.13)	0.039	3.69 (1.28–10.67)	0.016	3.32 (1.26–9.81)	0.015
Age, per year	1.02 (0.98–1.06)	0.382	1.01 (0.97–1.06)	0.550	1.01 (0.97–1.06)	0.544
Sex, female vs. male	1.41 (0.57–3.49)	0.458	1.48 (0.57–3.85)	0.418	1.43 (0.58–3.72)	0.439
Diabetes duration, per year	1.00 (0.95–1.06)	0.893	1.00 (0.94–1.07)	0.884	1.00 (0.95–1.07)	0.903
Preoperative NRS score, per point	0.88 (0.62–1.26)	0.494	0.76 (0.51–1.13)	0.174	0.77 (0.52–1.13)	0.188

Note: Pain response was coded as 1 and nonresponse as 0; ultrasound-positive status was coded as 1 and ultrasound-negative status as 0; female sex was coded as 1 and male sex as 0. Age, diabetes duration, and preoperative NRS score were modeled as continuous variables. All 107 patients were included in the conventional and Firth multivariable models. Firth confidence intervals are penalized profile-likelihood confidence intervals, and *p* values are based on penalized likelihood-ratio tests. OR, odds ratio; CI, confidence interval; NRS, numeric rating scale.

## Data Availability

The data supporting the findings of this study are available from the corresponding author upon reasonable request.

## Abbreviations

The following abbreviations are used in this manuscript:

ABI	Ankle–brachial index
ANCOVA	Analysis of covariance
BMI	Body mass index
CI	Confidence interval
CSA	Cross-sectional area
DN4	Douleur Neuropathique 4
DPN	Diabetic peripheral neuropathy
HbA1c	Glycated hemoglobin
HDL-C	High-density lipoprotein cholesterol
HRUS	High-resolution ultrasonography
IQR	Interquartile range
LDL-C	Low-density lipoprotein cholesterol
NRS	Numeric rating scale
OR	Odds ratio
PDPN	Painful diabetic peripheral neuropathy
SD	Standard deviation
